# Predicting Post-operative Blood Inflammatory Biomarkers Using Pre-operative Heart Rate Variability in Patients With Cervical Cancer

**DOI:** 10.3389/fphys.2021.696208

**Published:** 2021-11-04

**Authors:** Jian Liu, Shiqi Liu, Longfei Gao, Guangqiao Li, Jie Xu, Yilin Sun, Jingfeng Wang, Bo Shi

**Affiliations:** ^1^Department of Gynecologic Oncology, First Affiliated Hospital, Bengbu Medical College, Bengbu, China; ^2^School of Medical Imaging, Bengbu Medical College, Bengbu, China; ^3^Anhui Key Laboratory of Computational Medicine and Intelligent Health, Bengbu Medical College, Bengbu, China

**Keywords:** cervical cancer, heart rate variability, autonomic nervous, inflammatory biomarkers, Poincaré plot, frequency domain

## Abstract

Blood inflammatory biomarkers, including the neutrophil-to-lymphocyte ratio (NLR), the lymphocyte-to-monocyte ratio (LMR), and the platelet-to-lymphocyte ratio (PLR), play a significant role in determining the prognosis of patients with cervical cancer (CC). Currently, no methods are available to predict these indexes pre-operatively. Cardiac autonomic function is determined based on the heart rate variability (HRV), which is also associated with a progressive inflammatory response and cancer. Thus, the main aim of this study was to evaluate the feasibility of using pre-operative HRV parameters in CC patients to predict post-operative blood inflammation biomarkers as a means of determining prognosis. Between 2020 and 2021, 56 patients who were diagnosed with CC and then underwent hysterectomy surgery at the Department of Gynecologic Oncology, First Affiliated Hospital, Bengbu Medical College were enrolled in this study. Five-minute electrocardiogram data were collected 1 day before the operation for analysis of HRV parameters, including frequency domain parameters (LF, HF, and LF/HF) and Poincaré plot parameters (SD1, SD2, and SD2/SD1). Venous blood was collected 2 days post-operatively and inflammatory biomarkers were evaluated, with the NLR, LMR, and PLR determined. Pre-operative SD2 was significantly associated with post-operative PLR, with each 1-unit increase in SD2 decreasing the PLR value by 2.4 ± 0.9 (*P* < 0.05). Besides, LF/HF was significantly correlated with NLR, with each 1-unit increase in LF/HF increasing the NLR value by 1.1 ± 0.5 (*P* < 0.05). This association was independent of patient age and body mass index. These results suggest that the pre-operative autonomic nervous system plays a role in the regulation of post-operative cancer inflammation and that pre-operative HRV parameters can potentially predict post-operative inflammation and facilitate clinical treatment decisions.

## Introduction

Cervical cancer (CC) is a common gynecological malignancy, with its incidence and mortality ranked third and fourth, respectively, among female malignancies worldwide. Furthermore, CC seriously endangers a woman’s physical and mental health (Bray et al., 2018). Currently, the primary treatment for CC is a radical hysterectomy and chemotherapy, with about 10% of patients experiencing disease recurrence (Quinn et al., 2006; Greer et al., 2010). Moreover, the high heterogeneity that is present in tumor cells often contributes to the failure of traditional cancer therapies.

With the emergence of molecular biomarkers, targeted therapy, and other therapeutic techniques, cancer treatment approaches have transformed from evidence-based medicine to precise medicine (Ho et al., 2020). However, to improve clinical precision therapy decision-making, significant prognostic information is required (Gill, 2012). Currently, cancer prognosis assessments and therapeutic effect monitoring rely on blood tests, including hematological biomarker monitoring, and pathological analyses, with post-operative pathological analysis providing a meaningful basis for prognostic evaluation that can guide further treatment (Kasamatsu et al., 2009; Holdenrieder et al., 2016; Sopik and Narod, 2018; Han et al., 2021). Nevertheless, pathological analysis is a one-time analysis and cannot be used for dynamic cancer patient monitoring. In recent years, a number of studies have confirmed that CC patient prognosis is closely associated with blood inflammatory factors. Of these hematological indicators, neutrophil-to-lymphocyte ratio (NLR), lymphocyte-to-monocyte ratio (LMR), and platelet-to-lymphocyte ratio (PLR) have been shown to provide a reliable basis for evaluating CC patient prognosis and survival time (Trinh et al., 2020). However, post-operative blood inflammatory factors are often not predictable in advance. If the post-operative blood inflammatory factors in patients with CC can be predicted before surgery, they can provide more valuable information for the clinical decision-making of accurate treatment.

Clinical trials have demonstrated that heart rate variability (HRV), a non-invasive diagnostic tool for autonomic dysfunction, has a high specificity and positive predictive value in the prognostic assessment of cancer patients (Arab et al., 2016; Zhou et al., 2016; De Couck et al., 2018; Kloter et al., 2018). This is because autonomic nerves have a regulatory role in tumor gene expression, the surrounding microenvironment, and the inflammatory response (Cole et al., 2015; Gunterberg et al., 2016). Hu et al. (2018) demonstrated that HRV is closely associated with blood inflammatory factors, such as C-reactive protein (CRP) and interleukin (IL)-6, in patients with depression and anxiety disorders. Mouton et al. (2012) studied the relationship between HRV time-domain parameters and carcinoembryonic antigen (CEA) in 38 patients with colon cancer. They found that patients with low standard deviation of all normal-to-normal intervals (SDNN) (<20 ms) had significantly higher CEA at 1 year than patients with higher SDNN (≥20 ms), independent of confounders. The study showed that low HRV was associated with increased levels of tumor markers. De Couck et al. (2013) investigated the relationship between HRV time-domain parameters and prostate-specific antigen (PSA) in 113 patients with prostate cancer. They found that SDNN and the root mean square of successive interval differences (RMSSD) were negatively correlated with PSA levels at 6 months in prostate cancer patients, controlling for numerous confounders. In addition, RMSSD was also found to be negatively correlated with PSA levels at 2 years. Thus, HRV can inversely predict the level of tumor markers in cancer patients, which has important implications for early clinical intervention and prognosis evaluation of patients. However, pre-operative HRV has not been shown to predict post-operative inflammatory factor indicators such as LMR, NLR, or PLR in CC patients. In this study, pre-operative HRV was evaluated as a potential predictive factor to elucidate these inflammatory factors in patients with CC. It is hoped this pre-operative HRV detection can potentially guide post-operative CC clinical treatment in a non-invasive manner.

## Materials and Methods

### Subjects

The study cohort was comprised of CC patients (*n* = 60) who underwent hysterectomy surgery in the Department of Gynecology and Oncology, First Affiliated Hospital of Bengbu Medical College (Anhui, China) from November 2020 to January 2021. All patients met the following conditions: (i) CC diagnosed by pathological examination; (ii) treatment with hysterectomy surgery; (iii) no history of other malignant tumors; (iv) no primary diseases involving vital organs, such as heart, lung, liver, and kidney; (v) non-pregnant; (vi) no new adjuvant chemotherapy before surgery; and (vii) no missing clinical data. The study was approved by the Clinical Medical Research Ethics Committee of the First Affiliated Hospital of Bengbu Medical College (registration number: 2021KY010). All patients were informed of the details of the study, procedures, risks, and potential adverse effects of the experiment, and signed an informed consent form. The experimental procedures were performed in strict accordance with the ethical standards set forth in the 1964 Declaration of Helsinki and its amendments.

### Data Collection

A HeaLink R211B micro-electrocardiogram (ECG) recorder (Healink Ltd., Bengbu, China) was used 1 day prior to surgery to collect ECG data (5 min) from CC patients while in a supine position. During the ECG, the test environment was kept quiet and the patients were asked to relax and breathe smoothly. Measurements were obtained with disposable Ag/AgCl gel electrodes (JunKang Ltd., Shanghai, China). ECG signals were amplified on the device (bandwidth 0.67–40 Hz) and digitized with a 400 Hz sampling rate.

On post-operative day 2, median cubital vein blood was collected early in the morning, and neutrophil, lymphocyte, monocyte, and platelet counts were analyzed using a Sysmex XN hematology analyzer (Sysmex, Kobe, Japan). Based on the findings, LMR, NLR, and PLR values were calculated.

### Heart Rate Variability Analysis

The R-R interval (R-Ri) time series were extracted by ECG-Viewer software (HeaLink Ltd., Bengbu, China) with visual inspections afterward to remove artifacts (such as ectopic beats). Then, HRV frequency domain analysis and Poincaré plots quantitative analysis were performed.

### Frequency Domain Analysis

For the calculation of the power spectra, the R-Ri time series were resampled at 4 Hz using cubic spline interpolation. Subsequently, fast Fourier transform was applied. Power spectral density was calculated using Welch’s periodogram method with 150 s window width and 50% overlapping window. The low-frequency (LF; 0.04–0.15 Hz) power, the high-frequency (HF; 0.15–0.40 Hz) power, and the ratio of LF power to HF power (LF/HF) were used to evaluate HRV.

### Poincaré Plot Analysis

**FIGURE 1:**
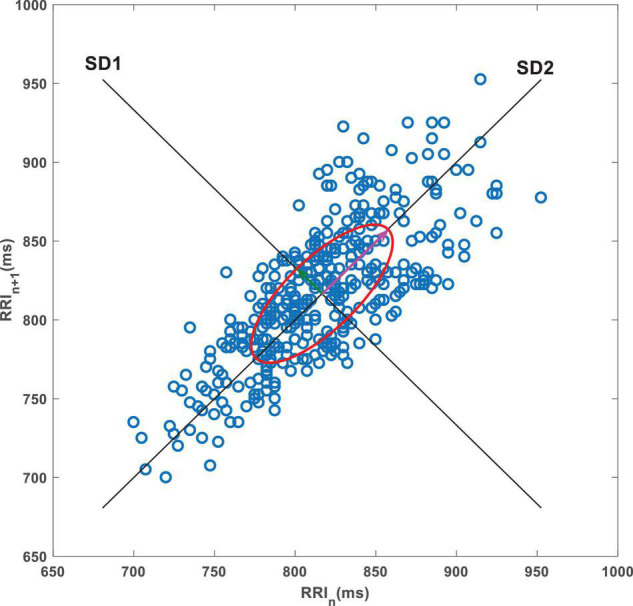
A representative Poincaré plot. The data are consecutive RRIs from a 5-min resting ECG recording from a CC patient. SD1, short axis of the ellipse; SD2, long axis of the ellipse.

Poincaré plot is a commonly used method in HRV non-linear analysis, which can directly show the pattern of heart rate fluctuation caused by non-linear activity. Herein, this plot was used to provide a graphical visualization of the R-Ri time series by capturing fluctuations in the intervals between heart beats (Figure 1). In this plot, each point represents an R-Ri that is correlated with the proceeding interval, with these points distributed across a Cartesian coordinate plane (Tulppo et al., 1996). Poincaré scatter plots can also provide qualitative visual analysis and quantitative analysis. The qualitative analysis results provide supplementary information for the standard time-domain analysis of HRV signals. Quantitative analysis is based on the distribution shape of the plot to extract mathematical features, and ellipse fitting is the most conventional method for quantitative analysis. Common ellipse metrics include short-axis and long-axis measurements, with the axis along the direction of the 45°contour of the ellipse being the long axis and the axis perpendicular to it being the short axis. These axes are commonly called semi-short (SD1) and semi-long (SD2) axes, where SD1 and SD2 are related to short-term and longer-term variability, respectively (Wei et al., 2020). The ratio of SD2 to SD1 (SD2/SD1) was considered as well.

### Statistical Analysis

The HF histogram showed a significant right skewed distribution; therefore, the HF was log transformed prior to analysis. A bivariate Pearson correlation analysis was used to determine potential correlations between the HRV parameters and the blood inflammatory markers. Conservatively, features with a *P* level of <0.1 were considered significant. A multiple linear regression analysis was then used to eliminate the effects of age and body mass index (BMI) with *P* < 0.05 deemed significant. All statistical analyses were performed using SPSS (ver. 23.0, IBM Corp., United States).

## Results

Cervical cancer cases (*n* = 60) were collected, with several cases excluded due to a lack of ECG data (*n* = 1), an excessive abnormal heart beat (*n* = 1), or due to abnormal data extremes (*n* = 2). The remaining CC cases (*n* = 56) were included in this study (age: 51.1 ± 10.6 years; BMI: 24.2 ± 3.1 kg/m^2^), and basic information and clinical data were obtained (Table 1). A bivariate Pearson’s correlation analysis was performed (Table 2), with PLR found to be positively correlated with LF/HF and negatively correlated with SD1, SD2, and HF (*P* < 0.05). Furthermore, NLR was positively correlated with both LF/HF and SD2/SD1 (*P* < 0.05), and LMR was positively correlated with SD1 (*P* < 0.05).

**TABLE 1:** Demographic, blood biomarker, and HRV CC patient data.

**Variables**	**Values**
*N* (Female)	56
Age (years)	51.1 (10.6)
BMI (kg/m^2^)	24.2 (3.1)
LMR	1.7 (0.8)
NLR	11.2 (5.0)
PLR	218.8 (85.5)
LF (ms^2^)	122 (88)
HF (ms^2^)	176 (209)
LF/HF	1.468 (1.284)
SD1 (ms)	12.4 (6.7)
SD2 (ms)	34.5 (12.2)
SD2/SD1	3.3 (1.5)

*Values are expressed as the number of patients, mean (standard deviation). Abbreviations: LF, low-frequency power; HF, high-frequency power; LF/HF, the ratio of low-frequency power to high-frequency power; NLR, the neutrophil-to-lymphocyte ratio; LMR, the lymphocyte-to-monocyte ratio; PLR, the platelet-to-lymphocyte ratio.*

**TABLE 2:** Bivariate correlation between HRV parameters and CC blood biomarkers.

	**LMR**	**NLR**	**PLR**
LF	(0.068, 0.620)	(0.056, 0.683)	(−0.192, 0.156)
HF	(0.184, 0.175)	(−0.181, 0.182)	**(−0.252, 0.061)**
LF/HF	(−0.165, 0.223)	**(0.305, 0.022)**	**(0.282, 0.036)**
SD1	**(0.238, 0.077)**	(−0.165, 0.225)	**(−0.239, 0.077)**
SD2	(0.147, 0.279)	(−0.041, 0.762)	**(−0.352, 0.008)**
SD2/SD1	(−0.212, 0.117)	**(0.234, 0.082)**	(0.082, 0.549)

*Values are expressed as (*r*, *P*). Bold indicates statistically significant at *P* < 0.1. Abbreviations: LF, low-frequency power; HF, high-frequency power; LF/HF, the ratio of low-frequency power to high-frequency power; NLR, the neutrophil-to-lymphocyte ratio; LMR, the lymphocyte-to-monocyte ratio; PLR, the platelet-to-lymphocyte ratio.*

**FIGURE 2:**
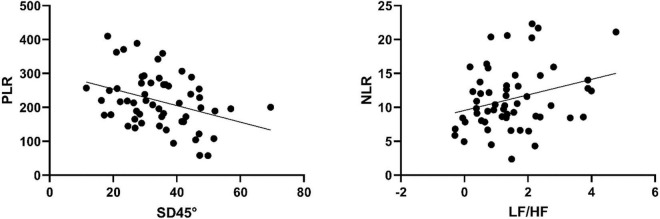
Partial correlation plot for HRV parameters and blood biomarkers after correcting for age and BMI.

To eliminate the confounding effects of age and BMI confounders, an additional multiple linear regression model was generated to eliminate these factors. The results showed that SD2 remained significantly correlated with PLR, with each 1-unit increase in SD2 decreasing the PLR value by 2.4 ± 0.9 (*P* < 0.05). Besides, LF/HF remained significantly correlated with NLR, with each 1-unit increase in LF/HF increasing the NLR value by 1.1 ± 0.5 (*P* < 0.05) (Table 3). To further examine this correlation, a partial correlation plot examining the outcome (PLR and NLR) and HRV parameters were generated (Figure 2).

**TABLE 3:** Results from the linear regression models (adjusted for age and BMI).

**Predictor**	**Outcome**	**Coefficient (Estimate ± SE)**	***P***
SD2	PLR	−2.4 ± 0.9	**0.009**
LF/HF	NLR	1.1 ± 0.5	**0.036**
SD1	LMR	0.04 ± 0.02	0.054
LF/HF	PLR	15.5 ± 9.3	0.101
SD1	PLR	−2.3 ± 1.8	0.200
SD2/SD1	NLR	0.6 ± 0.5	0.203
HF	PLR	−17.2 ± 14.7	0.246

*Bold indicates statistically significant at *P* < 0.05. Abbreviations: HF, high-frequency power; LF/HF, the ratio of low-frequency power to high-frequency power; NLR, the neutrophil-to-lymphocyte ratio; LMR, the lymphocyte-to-monocyte ratio; PLR, the platelet-to-lymphocyte ratio.*

## Discussion

The primary aim of this study was to determine if the pre-operative HRV parameters are correlated with post-operative blood inflammatory biomarkers (LMR, NLR, and PLR) in CC patients. The findings identified a negative correlation between the pre-operative SD2 and post-operative PLR, a positive correlation between the pre-operative LF/HF and post-operative NLR, and this association persisted after excluding both age and BMI following a multiple linear regression analysis. Overall, these results indicate that the use of pre-operative HRV to predict post-operative blood inflammatory markers in CC patients is promising.

Many studies have confirmed that autonomic nervous regulation is associated with the proliferation of inflammatory cells, including neutrophils, lymphocytes, and monocytes (Abo and Kawamura, 2002). Neutrophils and lymphocytes have adrenaline and acetylcholine receptors on their surfaces, which are regulated by both sympathetic and parasympathetic nerves. Specifically, when the sympathetic system is activated, the number of neutrophils increases, the number of lymphocytes decreases, and the value of NLR increases (Cao and Lawrence, 2002; Engler et al., 2004; Emeny et al., 2007). Conversely, when the parasympathetic nerve is excited, the number of neutrophils decreases, the number of lymphocytes increases, and the value of NLR decreases (Ono and Okada, 2013). LF/HF can be used to quantify the relationship between sympathetic and parasympathetic activity (Task Force of the European Society of Cardiology and the North American Society of Pacing and Electrophysiology, 1996). The increase in LF/HF is thought to reflect a shift to “sympathetic dominance,” while the decline in this index corresponds to “parasympathetic dominance.” The positive correlation between LF/HF and post-operative NLR in our study confirmed the regulatory effect of autonomic nerve on post-operative inflammation.

Platelet-to-lymphocyte ratio is considered a novel biomarker that reflects the inflammatory response and immune state of the body, as well as being closely related to the activation of the coagulation system (Zhao et al., 2018). SD2 quantitatively reflects the overall degree of heart rate variation, which represents the shared role of the sympathetic and vagus nerves (Shaffer and Ginsberg, 2017). Sympathetic activation promotes lymphocytopenia and can also promote bone marrow megakaryocyte proliferation, which subsequently increases the number of circulating platelets and the rate of platelet aggregation (Fu et al., 2019). These previous findings in conjunction with the physiological findings presented herein suggest the overall HRV mediated by sympathetic and vagus nerves has a negative association with the ratio of platelets to lymphocytes. However, the long-term effect of this association between the autonomic function and platelets is still unclear and will require further examination.

Current studies on multiple malignancies, including colorectal cancer (CRC), hepatocellular carcinoma (HCC), non-small cell lung cancer (NSCLC), ovarian cancer (OC), and CC, have shown that a high PLR is associated with a poor prognosis in cancer patients (Asher et al., 2011; Kwon et al., 2012). In CC studies, PLR has been shown to be significantly correlated with lymph node association and distant metastasis, as well as the response to radiotherapy (Zhang et al., 2014). Additionally, PLR can also be used as an independent International Federation of Gynecology and Obstetrics (FIGO) stage predictor (Zhu et al., 2018; Tas et al., 2019). Moreover, Tas et al. (2019) found that patients with invasive CC had a higher PLR when compared to a low-grade squamous intraepithelial lesion (LSIL). By contrast, a higher PLR is seen in high-grade squamous intraepithelial lesions (HSILs) when compared to patients with invasive CC, with elevated PLR and NLR both predictive of CC. In cancer patients, HRV has been shown to be significantly lower relative to healthy individuals, with levels also significantly lower in metastatic patients or patients with advanced stage III or IV when compared to non-metastatic patients (Bettermann et al., 2001; Nevruz et al., 2007; Lin and Chen, 2010; Koszewicz et al., 2016; Palma et al., 2016). Furthermore, in a study examining tumor markers in CRC patients, tumor marker levels were significantly increased in patients with a low HRV (Mouton et al., 2012). Wang et al. (2013) found that patients with brain metastases had significantly lower SDNN values relative to healthy individuals, with patients with an SDNN < 10 ms having a significantly lower survival than those with an SDNN > 10 ms. In another study investigating the relationship between HRV time-domain parameters and survival in pancreatic cancer, a higher SDNN (>20 ms) was significantly associated with a longer survival independent of confounding factors such as age or cancer treatment (De Couck et al., 2016). Previous studies have also shown that a high PLR and low HRV are indicative of a poor cancer prognosis, which is consistent with the findings presented herein.

At present, PLR, NLR, LMR, and other indicators can only be detected using invasive methods and cannot be predicted. In contrast, HRV analysis provides a non-invasive tool for assessing autonomic nervous system activity, usually by analyzing ECG time intervals or pulse wave signals (Kloter et al., 2018). Thus, the correlation between pre-operative HRV parameters and post-operative blood inflammatory factors identified herein tentatively provides a means to predict the prognosis of a CC patient pre-operatively and subsequently aid in guiding clinical treatment decisions.

Based on the above discussion, our study confirmed that it is feasible to predict post-operative blood inflammatory factors by examining pre-operative HRV values. However, this study also has some limitations. First, a previous study suggested that immune cell proportions and numbers follow a circadian rhythm and appear to be regulated by the autonomic nervous system (Suzuki et al., 1997). For example, during the day, the sympathetic nerve is active and the neutrophil numbers are increased. By contrast, at night, the vagus nerve is dominant and the lymphocyte numbers are increased. In this study, the samples were only collected in the morning; thus, different times of day could be optimal, and therefore other times should be explored in future work. Second, the potential long-term effects of autonomic function may lead to inflammatory level changes over time, which will require further investigating. Moving forward, it is necessary to study the dynamic fluctuations of HRV and inflammatory markers at different time points before and after surgery to understand the changes of autonomic nerve modulation over time during the inflammation process. Additionally, future studies should focus on examining a larger sample size and should consider more confounding factors.

## Data Availability Statement

The raw data supporting the conclusions of this article will be made available by the authors, without undue reservation.

## Ethics Statement

The study was approved by the Clinical Medical Research Ethics Committee of the First Affiliated Hospital of Bengbu Medical College (Registration Number: 2021KY010). The patients/participants provided their written informed consent to participate in this study.

## Author Contributions

BS and JL: conceptualization and resources. GL, JX, and YS: data collection. All authors: methodology. GL and JW: formal analysis. JL: data curation and supervision. BS and SL: writing—original draft preparation. BS, SL, and JL: writing—review and editing. BS: project administration and funding acquisition. All authors contributed to the article and approved the submitted version.

## Conflict of Interest

A direct family member of BS owns stock in HeaLink Ltd., Bengbu, China. The remaining authors declare that the research was conducted in the absence of any commercial or financial relationships that could be construed as a potential conflict of interest.

## Publisher’s Note

All claims expressed in this article are solely those of the authors and do not necessarily represent those of their affiliated organizations, or those of the publisher, the editors and the reviewers. Any product that may be evaluated in this article, or claim that may be made by its manufacturer, is not guaranteed or endorsed by the publisher.
